# Flavonoid-Rich Fruit Intake in Midlife and Late-Life and Associations with Risk of Dementia: The Framingham Heart Study

**DOI:** 10.14283/jpad.2024.116

**Published:** 2024-06-21

**Authors:** C. Lyu, P. F. Jacques, P. M. Doraiswamy, B. Young, A. S. Gurnani, R. Au, Phillip H. Hwang

**Affiliations:** 1 Department of Anatomy & Neurobiology, Boston University Chobanian & Avedisian School of Medicine, 72 East Concord Street, Boston, MA 02118 USA; 2 Jean Mayer USDA Human Nutrition Research Center on Aging, Tufts University, 711 Washington Street, Boston, MA 02111 USA; 3 Gerald J. and Dorothy R. Friedman School of Nutrition Science and Policy, Tufts University, 150 Harrison Avenue, Boston, MA 02111 USA; 4 Neurocognitive Disorders Program, Department of Psychiatry and Behavioral Sciences, Duke University School of Medicine, 905 West Main Street, Durham, NC 27701 USA; 5 Duke Center for the Study of Aging and Human Development, Duke University Medical Center, 201 Trent Drive, Durham, NC 27710 USA; 6 Duke Institute for Brain Sciences, Duke University School of Medicine, 308 Research Drive, Durham, NC 27710 USA; 7 Stanford University School of Medicine, 291 Campus Drive, Stanford, CA 94305 USA; 8 Departments of Neurology and Medicine, Boston University Chobanian & Avedisian School of Medicine, 72 East Concord Street, Boston, MA 02118 USA; 9 Framingham Heart Study, 73 Mount Wayte Avenue, Framingham, MA 01702 USA; 10 Department of Epidemiology, Boston University School of Public Health, 715 Albany Street, T3E, Boston, MA 02118 USA

**Keywords:** Dementia, fruit, flavonoids, midlife, late-life, Framingham Heart Study

## Abstract

**Background:**

Fruits are an important source of flavonoids, and greater intake of dietary flavonoids in older adults has been shown to be associated with decreased risk of dementia. It is unclear whether this relationship is similar or different between younger adults and older adults.

**Objectives:**

We examined for associations between midlife and late-life intake of flavonoid-rich fruits and incident dementia. We hypothesized that greater total cumulative intake of flavonoid-rich fruits in midlife and late-life adults would be associated with reduced risk of all-cause dementia.

**Design:**

Longitudinal, cohort study design.

**Setting:**

Framingham Heart Study, which is a longitudinal, multi-generational community-based cohort based in Framingham, Massachusetts, USA.

**Participants:**

Participants from the Framingham Heart Study Offspring cohort were included (n = 2,790) who attended the fifth core exam between 1991 to 1995, and were dementiafree and at least 45 years of age at that time, as well as had valid food frequency questionnaires from the fifth to ninth core exams.

**Measurements:**

Consumption of fruits with high flavonoid content or are important contributors to overall flavonoid intake was collected via food frequency questionnaire. Flavonoid-rich fruits from the food frequency questionnaire included raisins or grapes, prunes, bananas, fresh apples or pears, apple juice or cider, oranges, orange juice, grapefruit, grapefruit juice, strawberries, blueberries, and peaches, apricots, or plums. Dementia ascertainment was based on a multidisciplinary consensus committee, and included all-cause dementia and Alzheimer’s disease dementia diagnoses based on research criteria. Cox models were used to examine associations between cumulative fruit intake and incident dementia, stratified by midlife (45–59 years; n = 1,642) and late-life (60–82 years; n = 1,148).

**Results:**

Greater cumulative total fruit intake in midlife, but not late-life, was significantly associated with a 44% decreased risk of all-cause dementia (HR = 0.56; 95% CI = 0.32–0.98; p = 0.044). Decreased risk of all-cause dementia was also associated with higher intake of apples or pears in midlife and late-life, as well as higher intake of raisins or grapes in midlife only, and higher intake of oranges, grapefruit, blueberries, and peaches, apricots, or plums in late-life only.

**Conclusions:**

Among participants from the Framingham Heart Study, greater overall consumption of flavonoid-rich fruits in midlife was associated with reduced risk of dementia, though intake of specific fruits in midlife and late-life may have a protective role against developing dementia. These findings may help to inform future recommendations on when dietary interventions may be most beneficial to healthy brain aging across the life course.

**Electronic Supplementary Material:**

Supplementary material is available in the online version of this article at 10.14283/jpad.2024.116.

## Introduction

**I**ncreasing evidence suggests that a Mediterranean diet, which emphasizes flavonoid-rich fruits and vegetables, has the potential to reduce the risk of dementia, including Alzheimer’s disease (AD) dementia. (1–5). Flavonoids are naturally occurring bioactive pigments found widely in plant-based foods and have been studied for their potential neurocognitive benefits. (3, 4). Evidence from acute and short-term intervention studies on possible neurocognitive effects of flavonoid-rich foods is promising (6–13). A study from the Nurses’ Health Study (NHS) and the Health Professional Follow-Up Study found that long-term dietary flavonoid intake was associated with lower odds of subjective cognitive decline (14). Another study from the NHS showed that greater intake of blueberries and strawberries, which are one of the most common sources of flavonoids, was associated with slower rates of cognitive decline in older adults (15).

Studies that examine the relationship between intake of fruits high in flavonoids and risk of dementia are limited, however. Results from observational studies that have examined the relationship between flavonoid intake and dementia risk also reveal mixed results. Some studies failed to observe any associations between flavonoids and dementia, despite long follow-up periods (16, 17), while others have found a lower risk of dementia associated with higher flavonoid intake (18, 19). A recent study from the Framingham Heart Study (FHS) examining long-term dietary flavonoid intake found that higher flavonoid intake was associated with a 76% decreased risk of dementia in adults (20). This evidence suggests that fruits rich in flavonoids could potentially reduce the risk of developing dementia. Additional studies are therefore needed to systematically examine the impact of dietary fruit intake high in flavonoid content on dementia risk.

Another important limitation is that previous studies have not considered whether the association between dietary flavonoid intake from fruits and risk of dementia may differ by age. Most studies have focused on dietary flavonoid intake later in life (18, 21–24), but the potential benefits of flavonoids may begin earlier in life (25–27), including a study that found consumption of flavonoid-rich berries in young adults aged 20–30 years was effective in maintaining or improving cognitive performance (28). As recognition grows around dementia being a life course disease, with social and physiologic antecedents in early life (29), understanding the impact that flavonoid-rich foods can have across different time points in life on brain aging-related outcomes has important implications for maintaining cognitive function across the lifespan, and ultimately for delaying or potentially preventing the onset of dementia (30–32).

To address these limitations in previous studies examining associations between flavonoid-rich fruits and dementia risk, as well as extend previous work done at FHS by analyzing high-flavonoid fruit consumption measured earlier and later in life, we tested associations between total dietary intake of high-flavonoid fruits in midlife and late-life with incident all-cause dementia. We hypothesized that greater total intake of fruits high in flavonoid content during midlife and late-life is associated with decreased risk of all-cause dementia as compared to lower total intake of fruits high in flavonoid content.

## Methods

### Participants and procedures

Initiated in 1948, FHS is a population-based cohort with regular health exams (33). The present study used data from the FHS Offspring (Gen 2) cohort, which began in 1971–1975 with 5,124 participants who were 5–70 years of age and had, on average, health exams every four to eight years (34). To study the relationship between midlife and late-life consumption of flavonoid-rich fruits and dementia risk, analyses were based on 2,790 participants (Supplemental Figure 1) who (1) attended Exam 5 (1991–1995); (2) did not have dementia at Exam 5; (3) were at least 45 years of age at Exam 5; and (4) had valid food frequency questionnaires (FFQs) at Exams 5–9 (as described below). Informed consent was obtained from all study participants, and the study protocol was approved by the institutional review board of the Boston University Medical Campus.

### Dietary assessment

Participants’ dietary intakes were assessed at the fifth through ninth exams cycles (1991 - 2014) in the Gen 2 cohort using a validated semi-quantitative FFQ (35). Before each exam cycle, the FFQs were mailed to participants who were given instructions to complete the questionnaire by recording the frequency of foods consumed over the past 12 months, and to bring it to their exam appointment. The FFQ is comprised of a list of 126 foods with a standard serving size and a selection of nine frequency categories ranging from “never, or less than one serving per month” to “more than or equal to six servings per day.” A FFQ was judged as invalid if reported energy intakes were less than 600 kilocalories (kcal) per day or greater than 4000 kcal per day for women, and greater than 4200 kcal per day for men, respectively, or if more than 12 food items were left blank. Participants who had less than 12 blank items were included in the analysis and considered as non-consumers of the blank items. The validity of food intake measurements based on a comparison between the FFQ and two seven-day diet records collected during the time interval covered by the FFQ has been previously documented (36, 37).

The exposure of interest was dietary intake of fruits with high flavonoid content or fruits which are important contributors to overall dietary flavonoid intake. In the FFQ, there are a total of 15 fruit items included (raisins or grapes, prunes, bananas, cantaloupe, watermelon, fresh apples or pears, apple juice or cider, oranges, orange juice, grapefruit, grapefruit juice, other fruit juices, strawberries, blueberries, and peaches, apricots, or plums). Among these, we selected raisins or grapes, prunes, bananas, fresh apples or pears, apple juice or cider, oranges, orange juice, grapefruit, grapefruit juice, strawberries, blueberries, and peaches, apricots, or plums to be included in the analysis, based on their relatively high flavonoid content, or that they represent a major proportion of the total flavonoids consumed based on their consumption frequency (38, 39).

### Dementia ascertainment

Participants suspected of cognitive impairment are classified by expert consensus, including a team with at least one neurologist and one neuropsychologist, as to whether they have dementia (40). For this study, we followed participants in the study sample for the development of incident dementia over 28 years from the study baseline period. Diagnosis of all-cause dementia was based on the fourth edition of the Diagnostic and Statistical Manual of Mental Disorders (41). Diagnosis of AD dementia was based on the National Institute of Neurological and Communicative Disorders and Stroke and Alzheimer’s Disease and Related Disorders Association criteria (42).

### Covariates

Potential confounders considered in the analyses included age, sex, years of education, body mass index (BMI; kg/m^2^), physical activity index (PAI) expressed in metabolic equivalents (43), current smoking status (yes vs. no), prevalent stroke, hypertension, diabetes, apolipoprotein E (ApoE) ε4 allele carrier (at least one ε4 allele vs. no ε4 allele), and total energy intake (TEI; kilocalories per day).

Stroke was defined by the presence of any of the following events: atherothrombotic infarction of the brain, cerebral embolism, intracerebral hemorrhage, and subarachnoid hemorrhage (44). Hypertension was defined as having systolic/diastolic blood pressure ≥130/80 mmHg, or the use of anti-hypertensive medication for treating high blood pressure (45). Diabetes was defined as having non-fasted blood glucose ≥200 mg/dL, fasted blood glucose ≤126 mg/dL, or the use of insulin or hypoglycemic medication to lower blood sugar (46).

### Statistical analysis

Participants in the analytic sample (n = 2,790) were stratified into two age epochs, based on their age at Exam 5: midlife (n = 1,642, age 45–59 years); and late-life (n = 1,148, age 60–82 years). We provided descriptive statistics: mean and standard deviation for continuous variables, or count and percentage for categorical variables. When providing the distributions for the cumulative intake of total fruits and each fruit item included, we reported them as medians and interquartile ranges.

To examine associations between total fruit intake in midlife and late-life with incident all-cause dementia, we first calculated the cumulative average total fruit intake by updating the selected fruit items at each exam prior to censoring or until reaching the updating-stop rule for participants who developed dementia. Participants were followed from baseline until the occurrence of diagnosis of dementia, or death. They were censored at the dates at which these events occurred. Those who did not develop any of the above-mentioned events were followed up until the end of the study (December 31, 2019), at which time they were censored.

We updated total fruit intake at each exam by using the cumulative average of intakes of each fruit item from all exams prior to censoring, except as noted below. For participants diagnosed with dementia, we used a 365-day cutoff to determine when to stop updating their dietary data. If the difference between a participant’s dementia diagnosis date and the exam prior to dementia diagnosis was at least 365 days, we stopped the update at the most recent exam prior to the dementia diagnosis. However, if the difference was less than 365 days, then we stopped updating the dietary data at two exams prior to the exam at which dementia was diagnosed. For participants who died without developing dementia, we stopped updating their dietary data at the exam prior to their death. In the event that a participant was missing intake data from at least one of the follow-up exams, the cumulative average of total fruit intake was based on existing intake data. Finally, for participants who survived until the end of the study, we stopped updating their dietary information at the last exam (e.g., Exam 9). The same cumulative average approach was used to update the covariates at each exam, except for baseline age, sex, education, and ApoE genotype.

Separate Cox regression models were used to estimate hazard ratios (HRs) and 95% confidence intervals (CIs) for the association between total fruit intake in midlife and late-life, respectively, and incident all-cause dementia. We constructed models, unadjusted and adjusted for age, sex, education, BMI, physical activity, smoking, diabetes, hypertension, stroke, ApoE genotype, and TEI, that modeled total fruit intake as a binary variable (high, low) and categorical variable (highest, moderate, and lowest). Based on prior studies that found risk of dementia increased more dramatically in individuals with flavonoid intakes in the lowest 30% compared with higher intake levels (20), we compared participants at or below the 30th percentile (low) to those above the 30th percentile (high). As a categorical variable, we defined participants at the lowest 30th percentile as having lowest intake, followed by those between the 30th and 70th percentiles as having moderate intake, and finally, those at the 70th percentile or greater as having highest intake. We also conducted a sensitivity analysis to evaluate the impact of death as a competing event on the results from the primary analyses in the midlife and late-life groups.

Secondary analyses included: (1) examining independent associations with individual fruit intake in midlife and late-life, adjusting for intake of other specific fruit items; and (2) focusing on AD dementia as an outcome. The significance level was set to 0.05. All analyses were performed using Stata version 17.0 (StataCorp, College Station, Texas).

## Results

### Baseline characteristics of midlife and late-life participants & distribution of cumulative fruit intake

At baseline, the average age of the 2,790 participants (52% female) was 57.5 years (Table 1). Of these, 1,642 were in midlife (45–59 years) and 1,148 were in late-life (60–82 years) at study baseline. On average, participants in midlife were more likely to be female and individuals that smoke, as well as had more years of education and higher levels of physical activity compared to those in late-life. Participants in late-life were, on average, more likely to have hypertension, diabetes, and stroke compared to those in midlife.

**Table 1:** Baseline characteristics of the sample by midlife (45–59 years) and late-life (60–82 years) groups

	**Overall (n = 2,790)**	**Midlife (n = 1,642)**	**Late-life (n = 1,148)**
Age, years (mean (SD))	57.5 (8.1)	51.8 (4.2)	65.7 (4.4)
Sex (n (%))			
Female	1,461 (52.4)	874 (53.2)	587 (51.1)
Male	1,329 (47.6)	768 (46.8)	56 (48.9)
Education, years (mean (SD))	14.0 (2.6)	14.3 (2.6)	13.5 (2.6)
Body mass index, kg/m^2^ (mean (SD))	27.5 (4.9)	27.5 (4.9)	27.6 (4.8)
Physical activity index (median (IQR))	33.1 (30.2 – 36.6)	33.4 (30.6 – 37.1)	32.3 (30.1 – 35.8)
Current smoking (n (%))			
Yes	501 (18.0)	348 (21.2)	153 (13.3)
No	2,286 (81.9)	1,294 (78.8)	992 (86.4)
Hypertension (n (%))			
Yes	600 (21.2)	211 (12.8)	389 (33.9)
No	2,174 (77.9)	1,425 (86.8)	749 (65.2)
Diabetes (n (%))			
Yes	247 (8.8)	100 (6.1)	147 (12.8)
No	2,502 (90.0)	1,511 (92.0)	991 (86.3)
Stroke (n (%))			
Yes	274 (9.8)	100 (6.1)	174 (15.2)
No	2,516 (90.2)	1,542 (93.9)	974 (84.8)
ApoE ε4 allele carrier (n (%))			
Yes	627 (22.5)	378 (23.0)	249 (21.7)
No	2,163 (77.5)	1,264 (77.0)	899 (78.3)
Total energy intake, kcal (mean (SD))	1844.0 (617.1)	1857.2 (629.0)	1825.2 (599.4)

SD: Standard deviation; kg: Kilogram; m^2^: Meters squared; IQR: Interquartile range; ApoE: Apolipoprotein E; kcal: Kilocalories; Missing values include: n=345 for education; n=10 for body mass index; n=76 for physical activity; n=3 for smoking; n=16 for hypertension; n=41 for diabetes

Among the individual fruits examined, participants in the midlife group reported consuming, on average, fewer total fruits (11.1 servings/week) cumulatively compared to those in the late-life group (13.2 servings/week) (Table 2). The most commonly consumed fruit items in both groups were orange juice, bananas, and apples or pears, with consumption of these items being similar or higher among late-life participants compared to midlife participants.

**Table 2:** Distribution of median cumulative fruit intake by midlife and late-life groups

**Fruit item (servings per week)**	**Midlife**	**Late-life**
Total fruit intake (IQR)	11.1 (6.7 – 16.2)	13.2 (8.6 – 18.9)
Raisins or grapes (IQR)	0.4 (0.1 – 1.1)	0.5 (0.1 – 1.0)
Prunes (IQR)	0 (0 – 0.1)	0 (0 – 0.2)
Bananas (IQR)	1.7 (0.6 – 3.1)	2.5 (1.0 – 4.2)
Fresh apples or pears (IQR)	1.2 (0.5 – 2.5)	1.2 (0.5 – 3.0)
Apple juice or cider (IQR)	0.1 (0 – 0.5)	0.1 (0 – 0.5)
Oranges (IQR)	0.5 (0.2 – 1.4)	0.5 (0.2 – 1.7)
Orange juice (IQR)	2.0 (0.5 – 4.8)	3.5 (0.8 – 6.6)
Grapefruit (IQR)	0.2 (0 – 0.5)	0.2 (0 – 0.9)
Grapefruit juice (IQR)	0 (0 – 0.4)	0 (0 – 0.3)
Strawberries (IQR)	0.5 (0.2 – 1.0)	0.5 (0.2 – 0.7)
Blueberries (IQR)	0.3 (0.1 – 0.8)	0.2 (0 – 0.5)
Peaches, apricots, or plums (IQR)	0.3 (0.1 – 0.7)	0.5 (0.1 – 1.0)

IQR: Interquartile range

### Associations between total fruit intake in midlife and late-life with risk of dementia

Based on multivariable models adjusted for age, sex, education, BMI, physical activity, smoking, diabetes, hypertension, stroke, ApoE genotype, and total energy intake, high cumulative total fruit intake in the midlife group was significantly associated with decreased risk of all-cause dementia as compared to low cumulative total fruit intake (HR = 0.56; 95% CI = 0.32 – 0.98; p = 0.04) (Table 3). High cumulative total fruit intake in the late-life group was not significantly associated with decreased risk of all-cause dementia (HR = 1.10; 95% CI = 0.79 – 1.55; p = 0.56). High cumulative total fruit intake in midlife or late-life groups was not significantly associated with decreased risk of AD dementia (Supplemental Table 1). Accounting for death as a competing event did not meaningfully change the results, except that the association between high cumulative total fruit intake in the midlife group and decreased risk of all-cause dementia was nominally significant (p = 0.08) (Supplemental Table 2).

**Table 3:** Associations between cumulative total fruit intake in midlife and late-life groups and risk of all-cause dementia

**Total fruit intake percentile**	**Midlife (85 all-cause dementia cases/1,642 participants**		**Late-life (238 all-cause dementia cases/1,148 participants)**	
	**Hazard ratio (95% CI)**	**p-value**	**Hazard ratio (95% CI)**	**p-value**
Binary categorical				
≤30th percentile (Low)	1.00 (Reference)	—	1.00—(Reference)	—
>30th percentile (High)	0.72 (0.44 – 1.17)*	0.19	1.13 (0.85 – 1.52)*	0.40
	0.56 (0.32 – 0.98)†	0.04	1.10 (0.79 – 1.55)†	0.56
Three-level categorical				
≤30th percentile (Lowest)	1.00 (Reference)	—	1.00 (Reference)	—
>30th to 70th percentile (Moderate)	0.73 (0.42 – 1.25)*	0.25	1.24 (0.91 – 1.70)*	0.17
	0.57 (0.31 – 1.08)†	0.08	1.22 (0.86 – 1.74)†	0.26
>70th percentile (Highest)	0.71 (0.39 – 1.30)*	0.27	1.00 (0.71 – 1.40)*	0.98
	0.54 (0.26 – 1.09)†	0.09	0.90 (0.60 – 1.36)†	0.64

CI: Confidence interval; *Unadjusted model; †Adjusted for age, sex, education, body mass index, physical activity, smoking, diabetes, hypertension, stroke, ApoE genotype, and total energy intake

When examining potential dose-response relationships between cumulative total fruit intake in midlife and late-life groups with risk of all-cause dementia, we did not observe the highest intake category (HRmidlife = 0.54; 95% CImidlife = 0.26 – 1.09; pmidlife = 0.09; HRlatelife = 0.90; 95% CIlate-life= 0.60 – 1.36; plate-life = 0.64) nor moderate intake category (HRmidlife = 0.57; 95% CImidlife = 0.31 – 1.08; pmidlife = 0.08; HRlate-life = 1.22; 95% CIlate-life = 0.86 – 1.74; plate-life = 0.26) to be significantly associated with decreased all-cause dementia risk as compared with the lowest intake category (Table 3). Similar findings were also observed when examining potential dose-response relationships between cumulative total fruit intake in midlife and late-life groups with risk of AD dementia (Supplemental Table 1).

### Associations between individual fruits in midlife and late-life with risk of dementia

Decreased risk of all-cause dementia was independently associated with high cumulative intake of raisins or grapes (HR = 0.55; 95% CI = 0.32 – 0.95; p = 0.03), and apples or pears (HR = 0.56; 95% CI = 0.32 – 0.98; p = 0.04) in the midlife group (Table 4), after adjusting for intake of all other fruits included. We observed a nominally significant association between high cumulative intake of oranges in the midlife group and decreased risk of all-cause dementia (HR = 0.58; 95% CI = 0.34 – 1.00; p = 0.05). In the late-life group, decreased risk of all-cause dementia was independently associated with high cumulative intake of apples or pears (HR = 0.67; 95% CI = 0.50 – 0.90; p = 0.01), oranges (HR = 0.69; 95% CI = 0.52 – 0.93; p = 0.02), grapefruit (HR = 0.66; 95% CI = 0.49 – 0.88; p = 0.01), blueberries (HR = 0.66; 95% CI = 0.49 – 0.90; p = 0.01), and peaches, apricots, or plums (HR = 0.59; 95% CI = 0.43 – 0.79; p <0.01) (Table 4), after adjusting for intake of all other fruits included. Decreased risk of AD dementia was independently associated with high cumulative intake of apples or pears (HR = 0.66; 95% CI = 0.46 – 0.94; p = 0.02), grapefruit (HR = 0.69; 95% CI = 0.48 – 0.98; p = 0.04), blueberries (HR = 0.68; 95% CI = 0.47 – 0.97; p = 0.04), and peaches, apricots, or plums (HR = 0.52; 95% CI = 0.36 – 0.74; p <0.01) in the late-life group (Supplemental Table 3). Only high cumulative intake of raisins or grapes in the midlife group was nominally associated with decreased risk of AD dementia (HR = 0.50; 95% CI = 0.25 – 1.00; p = 0.05).

**Table 4:** Associations between cumulative individual fruit intakes in midlife and late-life groups and risk of all-cause dementia

	**Midlife**		**Late-life**	
Individual fruit intake (high vs. low)*	**Hazard ratio (95% Confidence interval)†**	**p-value**	**Hazard ratio (95% Confidence interval)†**	**p-value**
Raisins or grapes	0.55 (0.32 – 0.95)	0.03	0.77 (0.57 – 1.04)	0.09
Prunes	0.89 (0.49 – 1.61)	0.69	0.77 (0.58 – 1.04)	0.08
Bananas	1.16 (0.62 – 2.16)	0.64	0.94 (0.68 – 1.30)	0.70
Apples or pears	0.56 (0.32 – 0.98)	0.04	0.67 (0.50 – 0.90)	0.01
Apple juice or cider	1.10 (0.64 – 1.90)	0.73	0.93 (0.70 – 1.23)	0.62
Oranges	0.58 (0.34 – 1.00)	0.05	0.69 (0.52 – 0.93)	0.02
Orange juice	1.46 (0.77 – 2.77)	0.24	1.10 (0.80 – 1.52)	0.55
Grapefruit	1.10 (0.63 – 1.94)	0.74	0.66 (0.49 – 0.88)	0.01
Grapefruit juice	1.48 (0.86 – 2.53)	0.16	0.99 (0.75 – 1.31)	0.95
Strawberries	0.98 (0.55 – 1.76)	0.95	0.82 (0.60 – 1.11)	0.20
Blueberries	0.90 (0.50 – 1.63)	0.73	0.66 (0.49 – 0.90)	0.01
Peaches, apricots, or plums	0.79 (0.45 – 1.39)	0.42	0.59 (0.43 – 0.79)	0.001

*High group: >30th percentile; and Low group: ≤30th percentile (Reference); †Adjusted for age, sex, education, body mass index, physical activity, each specific fruit item, smoking, diabetes, hypertension, stroke, ApoE genotype, and total energy intake

When examining potential dose-response relationships with cumulative intake of specific fruit items, highest intake of raisins or grapes (HR = 0.35; 95% CI = 0.16 – 0.74; p = 0.01), apples or pears (HR = 0.51; 95% CI = 0.27 – 0.96; p = 0.04), and oranges (HR = 0.49; 95% CI = 0.24 – 0.98; p = 0.04) in the midlife group were independently associated with decreased risk of all-cause dementia (Table 5). We also found that highest intake of raisins or grapes (HR = 0.64; 95% CI = 0.45 – 0.90; p = 0.01), apples or pears (HR = 0.59; 95% CI = 0.42 – 0.83; p <0.01), grapefruit (HR = 0.59; 95% CI = 0.42 – 0.83; p <0.01), and blueberries (HR = 0.50; 95% CI = 0.34 – 0.74; p <0.01) in the late-life group were independently associated with decreased risk of all-cause dementia. Moreover, we observed that the cumulative intake of oranges in the late-life group at the highest (HR = 0.69; 95% CI = 0.50 – 0.97; p = 0.03) and moderate (HR = 0.70. 95% CI = 0.49 – 0.99; p = 0.04) categories, and with peaches, apricots, or plums (HRhighest = 0.51; 95% CIhighest = 0.35 - 0.74; phighest <0.01; HRmoderate = 0.64; 95% CImoderate = 0.46 – 0.89; pmoderate <0.01), were significantly associated with decreased risk of all-cause dementia. The association between highest levels of cumulative intake of prunes in the late-life group and risk of all-cause dementia was nominally significant (HR = 0.54; 95% CI = 0.29 - 0.99; p = 0.05). For associations with AD dementia risk (Supplemental Table 4), we found that only highest levels of cumulative intake of raisins or grapes in the midlife group (HR = 0.29; 95% CI = 0.11 – 0.78; p = 0.02) and in the late-life group (HR = 0.58; 95% CI = 0.38 – 0.88; p = 0.01), as well as highest levels of cumulative intake of apples or pears (HR = 0.60; 95% CI = 0.40 – 0.90; p = 0.01), grapefruit (HR = 0.55; 95% CI = 0.36 – 0.84; p <0.01), and blueberries (HR = 0.48; 95% CI = 0.30 – 0.76; p <0.01) in the late-life group were significantly associated with decreased risk of AD dementia. Both the highest (HR = 0.46; 95% CI = 0.30 – 0.72; p <0.01) and moderate (HR = 0.56; 95% CI = 0.38 – 0.84; p <0.01) levels of cumulative intake of peaches, apricots, or plums in the late-life group were significantly associated with decreased risk of AD dementia.

**Table 5:** Dose-response associations between cumulative individual fruit intakes in midlife and late-life groups and risk of all-cause dementia

	**Midlife**		**Late-life**	
Individual fruit intake (highest vs. moderate vs. lowest)*	**Hazard ratio (95% Confidence interval)†**	**p-value**	**Hazard ratio (95% Confidence interval)†**	**p-value**
Raisins or grapes				
Highest	0.35 (0.16 – 0.74)	0.01	0.64 (0.45 – 0.90)	0.01
Moderate	0.72 (0.40 – 1.30)	0.28	0.94 (0.67 – 1.32)	0.75
Prunes				
Highest	0.87 (0.34 – 2.24)	0.78	0.54 (0.29 – 0.99)	0.05
Moderate	0.89 (0.45 – 1.78)	0.75	0.84 (0.62 – 1.15)	0.28
Bananas				
Highest	0.93 (0.43 – 2.00)	0.86	0.85 (0.59 – 1.21)	0.36
Moderate	1.30 (0.68 – 2.48)	0.43	1.07 (0.74 – 1.55)	0.71
Apples or pears				
Highest	0.51 (0.27 – 0.96)	0.04	0.59 (0.42 – 0.83)	0.002
Moderate	0.64 (0.33 – 1.25)	0.41	0.78 (0.56 – 1.10)	0.16
Apple juice or cider				
Highest	0.94 (0.48 – 1.81)	0.84	0.89 (0.63 – 1.26)	0.52
Moderate	1.31 (0.69 – 2.50)	0.41	0.97 (0.69 – 1.35)	0.85
Oranges				
Highest	0.49 (0.24 – 0.98)	0.04	0.69 (0.50 – 0.97)	0.03
Moderate	0.65 (0.36 – 1.18)	0.16	0.70 (0.49 – 0.99)	0.04
Orange juice				
Highest	1.37 (0.68 – 2.73)	0.38	0.91 (0.64 – 1.30)	0.62
Moderate	1.60 (0.78 – 3.30)	0.20	1.42 (0.99 – 2.04)	0.06
Grapefruit				
Highest	0.87 (0.43 – 1.75)	0.69	0.59 (0.42 – 0.83)	0.002
Moderate	1.31 (0.71 – 2.42)	0.39	0.75 (0.53 – 1.07)	0.11
Grapefruit juice				
Highest	1.42 (0.70 – 2.92)	0.33	0.93 (0.62 – 1.39)	0.73
Moderate	1.50 (0.82 – 2.76)	0.19	1.03 (0.74 – 1.42)	0.87
Strawberries				
Highest	0.82 (0.39 – 1.71)	0.60	0.79 (0.55 – 1.14)	0.22
Moderate	1.08 (0.58 – 2.76)	0.19	0.84 (0.60 – 1.16)	0.29
Blueberries				
Highest	0.58 (0.27 – 1.28)	0.18	0.50 (0.34 – 0.74)	<0.001
Moderate	1.10 (0.60 – 2.03)	0.76	0.79 (0.57 – 1.09)	0.15
Peaches, apricots, or plums				
Highest	0.67 (0.33 – 1.36)	0.27	0.51 (0.35 – 0.74)	<0.001
Moderate	0.88 (0.48 – 1.63)	0.69	0.64 (0.46 – 0.89)	0.01

*Highest group: >70th percentile; Moderate group: >30th to 70th percentile; and Lowest group: ≤30th percentile (Reference); †Adjusted for age, sex, education, body mass index, physical activity, each specific fruit item, smoking, diabetes, hypertension, stroke, ApoE genotype, and total energy intake

## Discussion

The main finding of our study is that high cumulative intake of flavonoid-rich fruits starting in midlife was significantly associated with decreased risk of all-cause dementia. High cumulative intake of flavonoid-rich fruits in the late-life group was not associated with decreased risk of all-cause dementia. We did not observe dose-response relationships between cumulative intake of flavonoid-rich fruits in midlife or late-life groups and risk of all-cause dementia. This is one of the first studies to systematically examine for potential differences in associations between flavonoid-rich fruit intake in midlife and late-life individuals and incident dementia, which has important implications for developing more effective and timely prevention strategies for dementia.

Previous observational studies that have examined the relationship between total flavonoid intake and incident dementia have shown mixed results. Some studies found greater flavonoid consumption to be associated with decreased dementia risk (18, 20, 47, 48), but others did not observe an association between total flavonoid intake and dementia risk (16, 49, 50). Notably, most studies have only included participants and measurement of flavonoids in late-life (48–50). Very few studies included or exclusively focused emphasized adults in midlife or younger (20, 47), and those that included younger and older adults have not observed age to impact or modify the association between flavonoid intake and risk of dementia (20, 50). Other studies that focused on cognition as the outcome with a mixed age range of participants found that the association between greater total flavonoid intake and reduced cognitive decline was stronger in younger participants than older participants (14), though some did not find that age modified this association (12, 51–53).

Our findings that higher consumption of flavonoid-rich fruits, especially in the midlife group, was associated with decreased risk of all-cause dementia is consistent with hypothesized mechanisms of the beneficial neuroprotective effect of flavonoids. While much of their potential impact has been attributed to their antioxidant potential (54), either by means of their ability to scavenge reactive species or influence on intracellular redox status (55), flavonoids are more likely to exert favorable cognitive effects by protecting neurons from neurotoxins and combating neuroinflammation (56–58). Flavonoids and their metabolites may also result in favorable changes in cerebrovascular blood flow, which in turn can induce angiogenesis and neurogenesis (57, 59–61). It is believed that, through these proposed mechanisms, intake of flavonoid-rich foods, including fruits, over the lifespan may hold promise in delaying or possibly preventing the onset of dementia and age-associated cognitive decline.

We found that high intake of apples or pears starting in midlife and in late-life was significantly associated with decreased risk of all-cause dementia, while high intake of raisins or grapes starting in midlife and high intake of oranges, grapefruit, blueberries, and peaches, apricots, or plums starting in late-life was significantly associated with decreased risk of all-cause dementia. These findings should be interpreted cautiously, however, since we did not account for multiple testing as part of these secondary analyses. Published literature on the relationship between specific fruits and incidence of dementia is relatively limited, though a previous study of over 13,000 older adults found that consuming citrus fruits at least three to four times a week compared to two times or less a week was associated with a decreased risk of dementia (62). A meta-analysis including a sample of middle-aged and older adults found an inverse association between fruit and vegetable consumption and risk of cognitive impairment, though there was no significant association for individuals under 65 years of age (63). We also found that high intake of blueberries, as well as apples or pears, grapefruit, and peaches, apricots, or plums, in the late-life group was significantly associated with decreased risk of AD dementia. One study of mostly older adults found a significant association between increased consumption of berries and decreased AD dementia risk (64). Evidence from preclinical and animal studies is mixed, though, on whether blueberries and other flavonoid-rich fruits impact AD pathways, such as beta-amyloid accumulation or clearance (65–68). Further study is needed to confirm and extend our findings, especially in regards to examining the relationship between fruit consumption and incidence of dementia in younger adults.

Strengths of this study include the large sample sizes among midlife and late-life participants, cumulative average fruit intake assessment to reflect long-term impact, and long follow-up time for the occurrence of incident dementia cases. There are several limitations of this study that need to be considered. Our study focused on only midlife and late-life intake of flavonoid-rich fruits as data were limited or unavailable earlier in life, including early adulthood, as well as childhood and adolescence. We also acknowledge that consumption of flavonoid-rich fruits in late-life may be dependent or affected by consumption of flavonoid-rich fruits in midlife, which we could not examine in this analysis due to sample size limitations. Future studies should examine dietary factors across the entire life course, as well as evaluate changes in within-person consumption levels longitudinally across the life course and their associations with dementia risk. The FFQ intake data may be affected by misclassification, which likely would weaken our observed associations. To help address potential misclassification, we adjusted for total energy intake in our analyses. Our analyses were also limited in that we could not account for certain dementia risk factors because they were not available, such as depression. Therefore, unmeasured or residual confounding cannot be ruled out. Finally, participants in our study are primarily European American, which may limit generalizability of our findings to more ethnically diverse populations. Further study is needed to confirm our findings in more diverse study samples.

In conclusion, our findings are an important addition to the limited evidence that greater consumption of fruit, particularly starting in midlife, may be beneficial to brain aging by possibly reducing the risk of developing dementia later in life. These findings also highlight potential differences in the impact that fruit consumption may have on contributing to dementia risk across the life course. Future studies in larger and more age, as well as racially and ethnically, diverse individuals are warranted. With greater recognition of AD as a life course disease, more precise and effective prevention strategies are needed that emphasize the importance of tailoring interventions, such as diet, to the right person at the right time to promote brain health and delay or even potentially prevent the onset of dementia.

## Electronic supplementary material

Supplementary material, approximately 24.8 KB.

**Supplementary Figure 1**. Flowchart for selection of study participants

## Notes

*Ethical standards:* Informed consent was obtained from all study participants, and the study protocol was approved by the institutional review board of the Boston University Medical Campus.
