# Supplementary material for: Flavonoid-Rich Fruit Intake in Midlife and Late-Life and Associations with Risk of Dementia: The Framingham Heart Study
Source: J Prev Alzheimers Dis. 2024 Jun 21;11(5):1270–9. doi: 10.14283/jpad.2024.116 (PMC11436402; doi:10.14283/jpad.2024.116)
Supplement: Supplementary file 1 — Supplementary material, approximately 24.8 KB. [file 42414_2024_116_MOESM1_ESM.docx]

**Supplemental Table 1.** Associations between cumulative total fruit intake in midlife and late-life groups and risk of Alzheimer’s disease (AD) dementia

|  | **Midlife (52 AD dementia cases/1,642 participants** | | **Late-life (160 AD dementia cases/1,148 participants)** | |
| --- | --- | --- | --- | --- |
| **Total fruit intake percentile** | **Hazard ratio**  **(95% CI)** | **p-value** | **Hazard ratio**  **(95% CI)** | **p-value** |
| **Binary categorical** |  | |  | |
| **≤30^th^ percentile (Low)** | 1.00  (Reference) | -- | 1.00  (Reference) | -- |
| **>30^th^ percentile (High)** | 0.77  (0.41 – 1.44)* | 0.41 | 1.26  (0.87 – 1.81)* | 0.22 |
|  | 0.70  (0.34 – 1.44)^†^ | 0.33 | 1.10  (0.73 – 1.67)^†^ | 0.64 |
| **Three-level categorical** |  | |  | |
| **≤30^th^ percentile (Lowest)** | 1.00  (Reference) | -- | 1.00  (Reference) | -- |
| **>30^th^ to 70^th^ percentile (Moderate)** | 0.90  (0.43 – 1.88)* | 0.77 | 1.38  (0.94 – 2.03)* | 0.11 |
|  | 0.85  (0.36 – 2.05)^†^ | 0.72 | 1.20  (0.78 – 1.85)^†^ | 0.40 |
| **>70^th^ percentile (Highest)** | 0.68  (0.33 – 1.40)* | 0.29 | 1.11  (0.73 – 1.69)* | 0.63 |
|  | 0.60  (0.26 – 1.38)^†^ | 0.23 | 0.95  (0.58 – 1.54)^†^ | 0.83 |

CI: Confidence interval

*Unadjusted model

^†^Adjusted for age, sex, education, body mass index, physical activity, smoking, diabetes, hypertension, stroke, ApoE genotype, and total energy intake

**Supplemental Table 2.** Associations between cumulative total fruit intake in midlife and late-life groups and risk all-cause dementia, with death as a competing risk

|  | **Midlife (52 AD dementia cases/1,642 participants** | | **Late-life (160 AD dementia cases/1,148 participants)** | |
| --- | --- | --- | --- | --- |
| **Total fruit intake percentile** | **Hazard ratio**  **(95% CI)** | **p-value** | **Hazard ratio**  **(95% CI)** | **p-value** |
| **Binary categorical** |  | |  | |
| **≤30^th^ percentile (Low)** | 1.00  (Reference) | -- | 1.00  (Reference) | -- |
| **>30^th^ percentile (High)** | 0.59  (0.33 – 1.06)* | 0.08 | 1.10  (0.80 – 1.53)* | 0.55 |
| **Three-level categorical** |  | |  | |
| **≤30^th^ percentile (Lowest)** | 1.00  (Reference) | -- | 1.00  (Reference) | -- |
| **>30^th^ to 70^th^ percentile (Moderate)** | 0.62  (0.33 – 1.18)* | 0.15 | 1.12  (0.80 – 1.59)* | 0.51 |
| **>70^th^ percentile (Highest)** | 0.56  (0.27 – 1.15)* | 0.12 | 1.08  (0.72 – 1.60)* | 0.72 |

CI: Confidence interval

*Adjusted for age, sex, education, body mass index, physical activity, smoking, diabetes, hypertension, stroke, ApoE genotype, and total energy intake

**Supplemental Table 3.** Associations between cumulative individual fruit intakes in midlife and late-life groups and risk of Alzheimer’s disease (AD) dementia

|  | **Midlife** | | **Late-life** | |
| --- | --- | --- | --- | --- |
| **Individual fruit intake (high vs. low)*** | **Hazard ratio**  **(95% Confidence interval)^†^** | **p-value** | **Hazard ratio**  **(95% Confidence interval)^†^** | **p-value** |
| **Raisins or grapes** | 0.50  (0.25 – 1.00) | 0.05 | 0.74  (0.52 – 1.05) | 0.09 |
| **Prunes** | 0.74  (0.34 – 1.61) | 0.45 | 0.75  (0.53 – 1.06) | 0.10 |
| **Bananas** | 1.56  (0.67 – 3.63) | 0.30 | 0.92  (0.62 – 1.36) | 0.68 |
| **Apples or pears** | 0.67  (0.33 – 1.37) | 0.27 | 0.66  (0.46 – 0.94) | 0.02 |
| **Apple juice or cider** | 1.16  (0.58 – 2.28) | 0.67 | 1.00  (0.72 – 1.40) | 0.98 |
| **Oranges** | 0.53  (0.27 – 1.04) | 0.07 | 0.86  (0.60 – 1.24) | 0.43 |
| **Orange juice** | 1.47  (0.65 – 3.31) | 0.35 | 1.20  (0.81 – 1.77) | 0.35 |
| **Grapefruit** | 1.37  (0.66 – 2.83) | 0.40 | 0.69  (0.48 – 0.98) | 0.04 |
| **Grapefruit juice** | 1.39  (0.76 – 2.55) | 0.29 | 0.76  (0.54 – 1.07) | 0.11 |
| **Strawberries** | 1.20  (0.55 – 2.59) | 0.65 | 0.77  (0.54 – 1.12) | 0.17 |
| **Blueberries** | 0.93  (0.44 – 1.98) | 0.85 | 0.68  (0.47 – 0.97) | 0.04 |
| **Peaches, apricots, or plums** | 0.89  (0.44 – 1.83) | 0.76 | 0.52  (0.36 – 0.74) | <0.001 |

*High group: >30^th^ percentile; and Low group: ≤30^th^ percentile (Reference)

^†^Adjusted for age, sex, education, body mass index, physical activity, each specific fruit item, smoking, diabetes, hypertension, stroke, ApoE genotype, and total energy intake

**Supplemental Table 4.** Dose-response associations between cumulative individual fruit intakes in midlife and late-life groups and risk of Alzheimer’s disease (AD) dementia

|  | **Midlife** | | **Late-life** | |
| --- | --- | --- | --- | --- |
| **Individual fruit intake (highest vs. moderate vs. lowest)*** | **Hazard ratio**  **(95% Confidence interval)^†^** | **p-value** | **Hazard ratio**  **(95% Confidence interval)^†^** | **p-value** |
| **Raisins or grapes** |  | |  | |
| **Highest** | 0.29  (0.11 – 0.78) | 0.02 | 0.58  (0.38 – 0.88) | 0.01 |
| **Moderate** | 0.67  (0.32 – 1.40) | 0.29 | 0.95  (0.64 – 1.42) | 0.80 |
| **Prunes** |  | |  | |
| **Highest** | 0.69  (0.28 – 172) | 0.43 | 0.61  (0.30 – 1.21) | 0.15 |
| **Moderate** | 0.86  (0.25 – 1.72) | 0.80 | 0.79  (0.54 – 1.14) | 0.21 |
| **Bananas** |  | |  | |
| **Highest** | 1.33  (0.49 – 3.61) | 0.58 | 0.85  (0.55 – 1.30) | 0.45 |
| **Moderate** | 1.68  (0.70 – 4.05) | 0.24 | 1.03  (0.66 – 1.61) | 0.89 |
| **Apples or pears** |  | |  | |
| **Highest** | 0.54  (0.24 – 1.22) | 0.14 | 0.60  (0.40 – 0.90) | 0.01 |
| **Moderate** | 0.91  (0.40 – 2.11) | 0.83 | 0.73  (0.48 – 1.11) | 0.14 |
| **Apple juice or cider** |  | |  | |
| **Highest** | 1.13  (0.51 – 2.49) | 0.76 | 0.91  (0.60 – 1.39) | 0.67 |
| **Moderate** | 1.19  (0.52 – 2.74) | 0.68 | 1.09  (0.74 – 1.62) | 0.66 |
| **Oranges** |  | |  | |
| **Highest** | 0.51  (0.23 – 1.11) | 0.09 | 0.85  (0.56 – 1.30) | 0.46 |
| **Moderate** | 0.56  (0.24 – 1.29) | 0.18 | 0.87  (0.58 – 1.32) | 0.52 |
| **Orange juice** |  | |  | |
| **Highest** | 1.39  (0.62 – 3.11) | 0.43 | 0.94  (0.60 – 1.45) | 0.76 |
| **Moderate** | 1.73  (0.76 – 3.91) | 0.19 | 1.28  (0.85 – 1.94) | 0.23 |
| **Grapefruit** |  | |  | |
| **Highest** | 1.07  (0.44 – 2.60) | 0.88 | 0.55  (0.36 – 0.84) | 0.005 |
| **Moderate** | 1.64  (0.74 – 3.61) | 0.22 | 0.87  (0.58 – 1.31) | 0.52 |
| **Grapefruit juice** |  | |  | |
| **Highest** | 1.33  (0.67 – 2.65) | 0.42 | 0.69  (0.42 – 1.14) | 0.15 |
| **Moderate** | 1.50  (0.68 – 3.31) | 0.32 | 0.80  (0.54 – 1.18) | 0.26 |
| **Strawberries** |  | |  | |
| **Highest** | 1.08  (0.43 – 2.74) | 0.86 | 0.74  (0.48 – 1.15) | 0.18 |
| **Moderate** | 1.27  (0.56 – 2.92) | 0.57 | 0.80  (0.54 – 1.19) | 0.27 |
| **Blueberries** |  | |  | |
| **Highest** | 0.61  (0.23 – 1.64) | 0.33 | 0.48  (0.30 – 0.76) | 0.002 |
| **Moderate** | 1.12  (0.51 – 2.46) | 0.77 | 0.83  (0.57 – 1.22) | 0.35 |
| **Peaches, apricots, or plums** |  | |  | |
| **Highest** | 0.62  (0.25 – 1.58) | 0.32 | 0.46  (0.30 – 0.72) | 0.001 |
| **Moderate** | 1.11  (0.52 – 2.39) | 0.78 | 0.56  (0.38 – 0.84) | 0.005 |

*Highest group: >70^th^ percentile; Moderate group: >30^th^ to 70^th^ percentile; and Lowest group: ≤30^th^ percentile (Reference)

^†^Adjusted for age, sex, education, body mass index, physical activity, each specific fruit item, smoking, diabetes, hypertension, stroke, ApoE genotype, and total energy intake
