# Supplementary figures and images for: Flavonoid-Rich Fruit Intake in Midlife and Late-Life and Associations with Risk of Dementia: The Framingham Heart Study
Source: J Prev Alzheimers Dis. 2024 Jun 21;11(5):1270–9. doi: 10.14283/jpad.2024.116 (PMC11436402; doi:10.14283/jpad.2024.116)

Supplementary Figure 1. Flowchart for selection of study participants

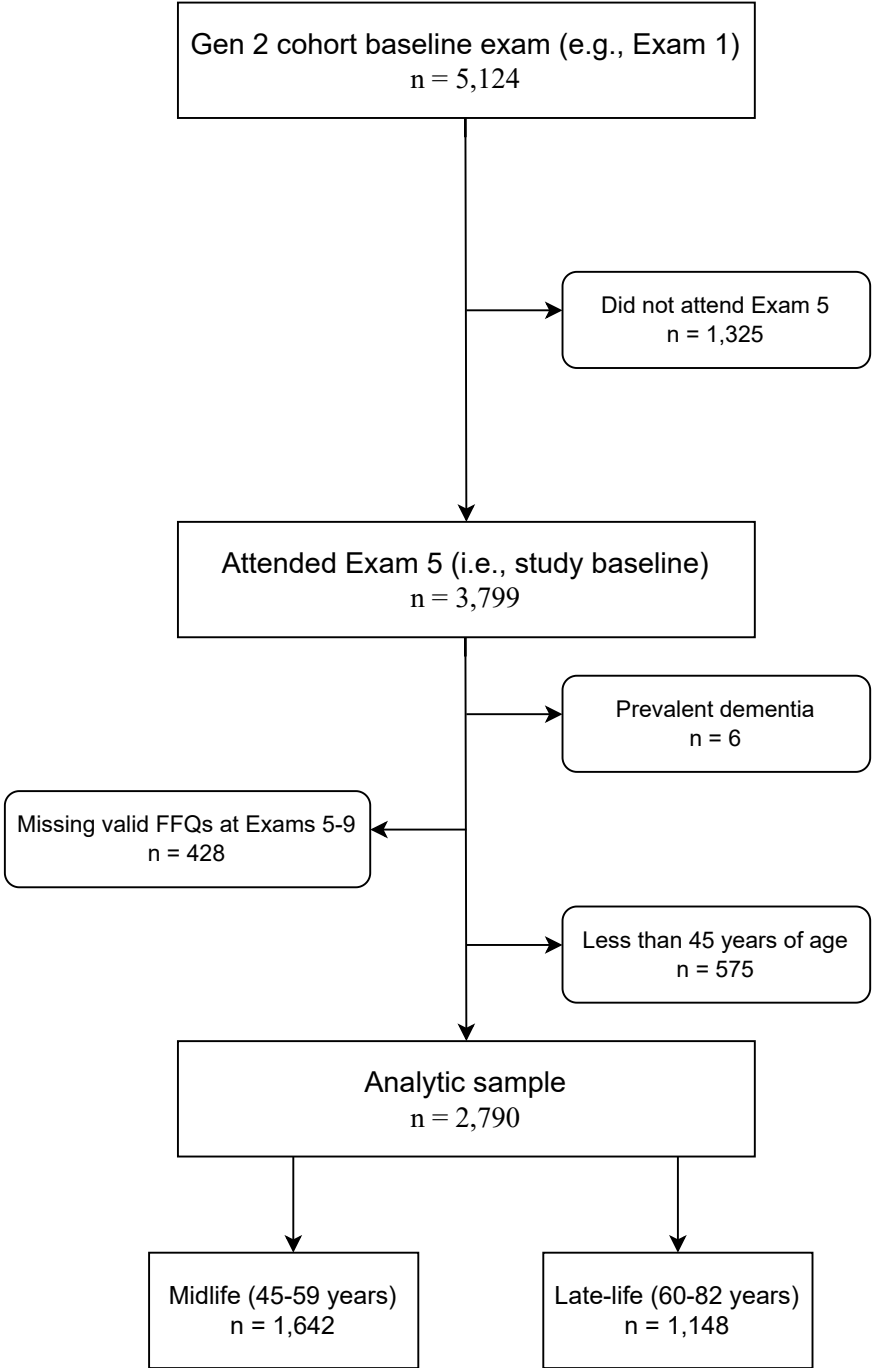

Supplement: Supplementary file 2 — Supplementary Figure 1. Flowchart for selection of study participants [file 42414_2024_116_MOESM2_ESM.pdf]
